# Hypertrichosis Is Not so Prevalent in Becker's Nevus: Analysis of 47 Cases

**DOI:** 10.1155/2014/953747

**Published:** 2014-04-30

**Authors:** Abbas Rasi, Hoda Berenji Ardestani, Seyed Mehdi Tabaie

**Affiliations:** ^1^Department of Dermatology, Hazrat-e Rasool University Hospital, Iran University of Medical Sciences, Tehran, Iran; ^2^Skin and Stem Cell Research Center, Tehran University of Medical Science, 4 Kamraniye Street, Maryam Alley, Tehran, Iran; ^3^Iranian Center for Medical Laser, Academic Center for Education, Culture and Research, Tehran, Iran

## Abstract

Becker's nevus is a relatively common cutaneous hamartoma, but is often overlooked or misdiagnosed. This nevus usually begins during the second decade of life as a circumscribed, hyperpigmented patch with irregular outline that gradually enlarges with associated hypertrichosis, developing several years later. The purpose of this study was to determine the prevalence of lesional hypertrichosis associated with Becker's nevus. *Methods*. 47 patients who had sharply demarcated brown patch with or without coarse hair, presence or enlargement of the lesion at the time of puberty, and compatible Wood's light examination were enrolled. Patients who had axillary freckling, previous skin inflammation, and gray pigmentation of the lesions were excluded. *Results*. In summary, the mean age of onset was 11.89 (range 0–19). The most commonly involved site was the arm (34%), followed by shoulder (23.4%), chest, face, flank, buttock, and leg. Lesional hypertrichosis was found in only 8 (17%) of the 47 patients. In 29 cases (61.7%) the lesions were in the right side of the body. *Conclusion*. Hypertrichosis was not so frequent among patients with Becker's nevus. There was a higher preponderance of the lesions on the right side.

## 1. Introduction

In 1949, Becker first described two cases of a hyperpigmented and hypertrichotic skin lesion with unilateral arrangement [1]. Becker's nevus is a relatively common cutaneous hamartoma but is often overlooked or misdiagnosed [2]. Since Becker's original article, many more cases have been reported. The lesion has also been called pigmentary hairy epidermal nevus [3]. Because of its androgen dependency, the lesion may present in a less conspicuous fashion in women with less intense pigmentation and mild, or even absent, hypertrichosis [4], leading to the failure of a diagnosis of Becker's melanosis. The hyperpigmentation usually remains stable, although there have been reports of fading over many years [5, 6]. This nevus usually begins during the second decade of life as a circumscribed hyperpigmented patch with irregular outline, which gradually enlarges with associated hypertrichosis, developing several years later. It is an androgen-dependent lesion because it becomes more prominent after puberty and tends to be more conspicuous in male patients because of an increased hairiness of this area [7]. The intralesional presence of acne and the occurrence of this nevus in a patient with accessory scrotum further reflect the pathogenic role of androgens in this disorder [7–15]. An increased number of androgen receptors, as compared with unaffected skin, have been reported [7, 16], initially pale in color and becoming more conspicuous after sun-exposure, with new macules developing beyond the margin and fusing with it, giving a geographical contour.

## 2. Materials and Methods

A total of 47 patients with the typical features of Becker's nevus were selected from 22,600 patients attending the outpatient dermatology department in Hazrat-e Rasool Hospital, Tehran, Iran, from July 2003 to July 2010. Patients were briefed on the details of the study and informed consent was obtained from subjects who agreed to participate. The study was approved by the research ethics committee of the Tehran University of Medical Sciences. All subjects were examined by two trained dermatologists. Each patient was subjected to a detailed review of clinical history and a complete physical examination including the skin. Examination was also conducted to define anatomic location, hairiness of the lesions with a magnifying lens compared with control unaffected skin, family history, sex, and age of onset.

### 2.1. Inclusion Criteria

Patients with the following criteria were included in the study: (1) presence of sharply demarcated brown patch with or without coarse hair, (2) presence or enlargement of the lesion at the time of puberty, and (3) Wood's light examination to rule out melanocytic lesions (such as congenital melanocytic nevus and nevus spilus).

### 2.2. Exclusion Criteria

Patients who had axillary freckling, previous skin inflammation, and gray pigmentation of lesions were excluded from the study.

## 3. Result

In our study among 22,600 male and female patients between the ages of 12 and 42, the frequency of Becker's nevus was 0.2%. The 47 patients had a mean age of 17.1 years (range: 12–42 years). There were 33 male patients (70.2%), with a mean age of 17 years (range: 12–42 years) and 14 female patients (29.8%), with a mean age of 22.85 years (range: 15–29 years). In summary, the mean age of onset was 11.89 years (range: 0–19). The most frequent age of onset was 12 years (53.2% of cases). The most commonly involved site was the arm (34%), followed by shoulder (23.4%), chest (17%), face (6.5%), flank (6.5%), buttock (4.2%), and leg (4.2%) (Table 1). Lesional hypertrichosis was found in only 8 (17%) of the 47 patients; of these 6 were male and 2 were female (Table 2).

Family history was positive in 3 cases (6.38%) and all of them were female (*P*value = 0.005). In 29 cases (61.7%), the lesions were in the right side of the body. Prominent smooth muscle hamartoma (SMH) with accompanying vermicular movements and pillar erection on palpation was a conspicuous finding in only 3 patients (6.38%). These patches frequently showed follicular accentuation. These patients were two males and one female and 18, 28, and 42 years old, respectively.

## 4. Discussion

Since Becker's original article, many more cases have been reported. Becker's nevus has been described in all races [17]. Tymen et al. [18] reported the frequency of the lesion to be 0.52% in a survey of 19,302 French military male recruits between the ages 17 and 26. In another survey among 1,146 school children, the prevalence of Becker's nevus was 2% [19]. In another study, Becker's nevus was found in 0.25% of 28,000 Italian men enlisted for the navy [20]. In our study among 22,600 male and female patients between the ages of 12 and 42, the frequency of BN was 0.2%. Becker's nevus is an androgen-dependent lesion because it becomes more prominent after puberty and tends to be more conspicuous in male patients because of an increased hairiness of this area [7]. Hairiness becomes eminent with age or can be absent or very slight [21]. The 47 patients had a mean age of 17.1 years (range: 12–42 years). There were 33 male patients (70.2%) with a mean age of 17 years (range: 12–14 years) and 14 female patients (29.8%) with a mean age of 22.85 years (range: 15–29 years).

However, Happle and Koopman suggested that the true sex ratio may in fact be 1 : 1 because this nevus tends to be less conspicuous in female patients [4]. In another study it was four, five, and six times more common in males than females [6, 18, 22]. In our study, however, there was a higher male preponderance with a male to female ratio of 2.3 : 1.

This nevus usually begins during the second decade of life as a circumscribed, hyperpigmented patch with irregular outline that gradually enlarges with associated hypertrichosis, developing several years later.

There were 33 male patients, with a mean age of onset 12.52 years, and 14 female patients, with a mean age of onset 10.43 years. As a result, the mean age of onset was 11.68 (range: 0–19). A bimodal distribution was identified with the onset of disease: congenital (1 female, 1 male) and 3 to 17 years (32 males, 13 females). When the age at onset was compared between male and female patients, no significant differences were found (*P*value = 0.296). In the series reported by [20], the age at onset was 5–17 years, mean 11.9 years.

In male patients, the lesion may develop increased hairiness after puberty [9]. Hypertrichosis usually develops after the hyperpigmentation, and the hairs become coarser and darker with time. In our hands, lesional hypertrichosis was found in 8 (17%) of the 47 patients. In this study, we observed that the hypertrichosis was not so prevalent among patients with Becker's nevus, since nearly 87% of our patients did not have this sign. By contrast, increased terminal hairs were observed in 70% of the Italian cases [20]. In the survey of 19,302 males recruited by Tymen et al., hypertrichosis was present in only a little more than 50% of Becker's lesions [18]. One possible explanation for the lower number of hypertrichosis cases in our study is that the mean age of our patients was lower than in these studies. When the age at onset was compared between male and female patients, no significant differences were found (*P* = 0.296). We believe that cases similar to ours are common but have been labeled as other skin disorders. Rower et al. [23] reported 5 cases of progressive cribriform and zosteriform hyperpigmentation. In all 5 cases, the age of onset was during the peripubertal or adolescent years. None of the lesions had associated hypertrichosis. We agree with Patrizi et al. [24] that these reports are most likely cases of Becker's melanosis without hypertrichosis.

The most commonly involved site was the arm (34%), followed by shoulder (21.3%), chest (17%), face (6.4%), flank (6.4%), buttock (4.3%), and leg (4.3%), with 45 patients having upper trunk involvement (95.7%) and only 2 patients having lesions limited to the lower trunk (4.3%). Our data confirms the findings of previous studies that Becker's nevus is more frequent in upper trunk [25, 26]. Involvement of the right and left side of the body was 29 (61.7%) and 18 (38.3%), respectively. This has not been reported previously.

The incidence of smooth muscle hamartoma (SMH) in Becker's melanosis is difficult to determine because of the lack of a general agreement on the criteria for Becker's melanosis. Prominent SMH with accompanying vermicular movements and pillar erection on palpation was a conspicuous finding in only 3 patients (6.4%). These patches frequently showed follicular accentuation.

Most Becker's nevi occur as isolated defects; however, ipsilateral bony abnormalities, acneiform eruptions [11], and breast hypoplasia have been reported [22] in patients with Becker's nevi. Hypoplasia may involve the entire breast or only the nipple and areola. In female patients, this is the most frequently reported anomaly to be associated with Becker's nevus [2, 13, 16, 27]. We did not show any associated physical and developmental abnormality. But in our study only two female patients had ipsilateral breast hypoplasia.

Although it is usually acquired, some cases are congenital. Becker's nevus has been reported in siblings [28], father and son [29], and uncle and nephew [30]. In our study positive family history was present only in 6.4% of patients. But the information on family history may be unreliable because no confirmation of diagnoses was possible and the data may be subject to recall bias.

Becker's nevus is a benign condition and there have been no reports of malignant transformation. Once established, it remains for the rest of one's life.

The hyperpigmentation has been successfully treated with Q-switched ruby and frequency-doubled Nd:YAG but recurrence rates are high [31]. In a comparative study by [32] for pigment removal, one pass with erbium:YAG was superior to three treatment sessions with Nd:YAG. In another study by [33], fractional resurfacing with 1550 nm erbium-doped fiber laser, more than 75% of pigment had faded by one month. There was no improvement in hypertrichosis.

Skin camouflage advice can be helpful [34]. Traditional surgical approaches either are unsuccessful or result in significant scarring. Laser technology offers the clinician a means to reduce both the pigmentation and the hypertrichosis often seen in Becker's nevus and therefore may improve the cosmetic appearance of the lesion. Electrolysis is a well-established method of epilation but its use in removing hair from Becker's nevus has not been described. Corrective makeup with a variety of water-resistant and light to very opaque products may be a valid adjunctive therapy for patients undergoing long-term treatment or in whom conventional therapy is ineffective.

## 5. Conclusion

To our knowledge, this is the largest series of patients with Becker's nevus from Iran. Two unexpected clinical findings were noted in this study: (1) hypertrichosis being not so frequent among patients with Becker's nevus, since nearly 87% of our patients did not have this sign, and (2) higher preponderance of the lesions on the right side. Involvement of the right and left side of body was 29 (61.7%) and 18 (38.3%), respectively. This has not been reported previously.

## Figures and Tables

**Table 1:** 

					Count					
					Place					Total
	Arm	Shoulder	Chest	Face	Flank	Buttock	Forearm	Leg	Back
Gender										
Female	5	3	3	1	1	1	0	0	0	14
Male	11	7	5	2	2	1	1	2	1	33
Total	**16**	**10**	**8**	**3**	**3**	**2**	**1**	**2**	**1**	**47**

**Table 2:** 

	Hypertrichosis		Total
	No	Yes	
Gender			
Female	11	3	14
Male	28	5	33
Total	**39**	**8**	**47**
